# Gating Behavior of Endoplasmic Reticulum Potassium Channels of Rat Hepatocytes in Diabetes

**DOI:** 10.6091/ibj.1308.2014

**Published:** 2014-07

**Authors:** Maedeh Ghasemi, Naser Khodaei, Sajjad Salari, Afsaneh Eliassi, Reza Saghiri

**Affiliations:** 1 *Neurophysiology Research Center, Shahid Beheshti University of Medical Sciences, Evin, Tehran 19857, Iran; *; 2 *Dept. of Physiology, Shahid Beheshti University of Medical Sciences, Evin, Tehran 19857, Iran; *; 3 *Dept. of Physiology, Ilam University of Medical Sciences, Ilam, Iran;*; 4 *Neuroscience Research Center, Shahid Beheshti University of Medical Sciences, Evin, Tehran 19857, Iran;*; 5 *Dept. of Biochemistry, Pasteur Institute of Iran, Tehran, Iran*

**Keywords:** Endoplasmic reticulum, Diabetes, Ion channels, Bilayer lipid membrane, Liver

## Abstract

**Background: **Defects in endoplasmic reticulum homeostasis are common occurrences in different diseases, such as diabetes, in which the function of endoplasmic reticulum is disrupted. It is now well established that ion channels of endoplasmic reticulum membrane have a critical role in endoplasmic reticulum luminal homeostasis. Our previous studies showed the presence of an ATP-sensitive cationic channel in endoplasmic reticulum. Therefore, in this study, we examined and compared the activities of this channel in control and diabetic rats using single-channel recording techniques. **Method: **Male Wistar rats were made diabetic for 2 weeks with a single dose injection of streptozotocin (45 mg/kg). Ion channel incorporation of rough endoplasmic reticulum of diabetic hepatocytes into the bilayer lipid membrane allowed the characterization of K^+^ channel. **Results: **Ion channel incorporation of rough endoplasmic reticulum vesicles into the bilayer lipid revealed that the channel current-voltage (I-V) relation with a mean slope conductance of 520 ± 19 pS was unaffected in diabetes. Interestingly, the channel Po-voltage relation was significantly lower in diabetic rats at voltages above +30 mV. **Conclusion: **We concluded that the endoplasmic reticulum cationic channel is involved in diabetes. Also, this finding could be considered as a goal for further therapeutic plans.

## INTRODUCTION

Endoplasmic reticulum is a major homeostatic subcellular compartment that fulfills multiple cellular functions, including calcium homeostasis, lipid synthesis, protein folding, quality control of newly synthesized proteins, and drug detoxification [1]. Perturbation of endoplasmic reticulum homeostasis can eventually trigger injury and cell apoptosis and leads to a disease [2, 3]. 

Recent studies have provided evidence that mitochondrial and endoplasmic reticulum dysfunction are major factors in two pathological arms (peripheral insulin resistance and defective insulin secretion) of diabetes and its complications, including beta-cell failure, endothelial dysfunction, cardiomyopathy, nephropathy, and neuropathy [4-7]. 

The normal function of these organelles (mitochondrial and endoplasmic reticulum) is tightly regulated by interdependent processes, such as the activation of ion channels, exchangers, pumps, and other proteins on their membrane. Indeed, intracellular ion channels have been recognized as an important contributor to cellular homeostasis and health maintenance by controlling the ion currents and potential across intracellular membrane, electro neutrality, pH, and organelle volume [8]. Disturbances in one of these processes will profoundly influence the other that can be the onset step of pathogenesis of a disease. Diabetic heart abnormalities occur mainly due to defects in sarcolemma Na-K-ATPase, Na-Ca^2+^ exchanger, Na-H exchanger, Ca^2+^-pump, and Ca^2+^ channels activities as well as due to changes in sarcoplasmic reticular Ca^+2 ^uptake and Ca^+2^ release processes. These observations suggest that sarco-plasmic reticulum function in diabetic heart may be defective as well as contributory to depressed cardiac performance in chronic diabetes [9]. Our previous studies described the presence of an ATP- and voltage-sensitive cationic channel in rough endoplasmic reticulum of rat Hepatocytes [10]. Because endoplasmic reticulum potassium channels have been involved in several functions, including protein folding, apoptosis, and calcium homeostasis, a study was undertaken to investigate if the gating behavior of the cationic channel was altered in a diabetic model. Pathophysiological roles for K^+^ channels have been shown in numerous studies related to diabetic cell/organelle injury [11, 12]. For example, reduction of kinetic properties and molecular composition of Kv, Kir, K_ATP_, and BK channels and also decrease of potassium current have been proposed to increase the cardiac action potential and to reduce dilation of coronary artery and aorta, which are consistently observed in diabetic condition [13-15]. Furthermore, it has been demonstrated that mitochondrial potassium channel displays abnormalities in diabetic heart [16]. Therefore, identifying the behavior aspects of endoplasmic reticulum cation channel in a diabetic model will provide new insights into our understanding of cellular mechanism of diabetes.

## MATERIALS AND METHODS

HEPES, Trizma Base (2-amino-2-[hydroxymethyl]-1,3-propanediol), sucrose, potassium chloride, ATP, ethylene glycol tetraacetic acid, and glibenclamide were purchased from Sigma (St. Louis, MO, USA) and n-Decane was obtained from Merck (Darmstadt, Germany). Salt and solvent were analytical grade (Sigma, St. Louis, MO, USA).

***Induction of experimental diabetes in rats. ***Animal experiments were conformed according to the National Institutes of Health Guidelines for the Care and Use of Laboratory Animals and approved by the Animal Ethics Committee of Shahid Beheshti University of Medical Sciences (Tehran, Iran). Two-month-old male Wistar rats, weighing 200–220 g, were allowed to acclimatize for seven days in an environmentally controlled room at 22°C with an alternating 12-h light/dark cycle and free access to normal laboratory food and water. After one-week acclimation, the animals were randomly assigned to either control or diabetic groups. Rats in the control group were continually fed only normal laboratory food. Diabetic rats were prepared by giving an intraperitoneal injection of 45 mg/kg streptozotocin (STZ, Sigma-Aldrich, Saint Louis, Missouri, USA) dissolved in 0.1 M citrate-buffered saline (pH 4.5) into the fasting rats. Serum glucose levels were checked on day 0 (before STZ injection) and also 7 and 14 days after STZ administration. Animals were considered to be diabetic if they had plasma glucose concentration of 250 mg/dl or greater in addition to polyuria and other diabetic features [17]. All studies were carried out two weeks after the injection of STZ or saline (control group). After two weeks, control and diabetic rats were euthanized by diethyl ether, and the livers were excised for the study.

***Lipid preparation.*** *L-*α*-*phosphatidylcholine (L-α-lecithin) was extracted from fresh egg yolk by the procedure described by Singleton *et al.* [18]. The endoplasmic reticulum membrane was relatively enriched in the neutral zwitterionic phospholipids having large polars head groups such as L-α-phosphatidylcholine [19, 20]. 

***Endoplasmic reticulum isolation. ***Hepatic endoplasmic reticulum vesicles from control and diabetic rats were separately isolated by the method described previously [21] with minor modifications. Briefly, rats were anesthetized by ether, and the livers were rapidly removed and homogenized in 50 ml ice-cold sucrose (0.25 M) solution at 2850 rpm using a potter homogenizer (Potter-Elvehjem Homogenizer, Iran). The homogenate was centrifuged at 8700 ×g for 13 min. The supernatant was centrifuged at 110,000 ×g at 4°C for 60 min (Beckman model J-21B, USA). The pellet was gently resuspended in 9 ml ice-cold 2 M sucrose by a glass homogenizer to obtain a homogenous suspension. Subsequently, in sucrose gradient conditions, the suspension was centrifuged at 300,000 ×g for 60 min, and the obtained pellet was dissolved in 20 ml sucrose 0.25 mM + imidazole 3 mM + Na pyrophosphate 0.5 mM. The solution was then centrifuged three times at 140,000 ×g for 40 min. The obtained pellet (rough endoplasmic reticulum microsomes microsomes) was dissolved in 1 ml sucrose 0.25 mM + imidazole 3 mM at a final concentration of 7 mg/ml. Rough microsomes were stored in 10-µl aliquots in 250 mM sucrose/3 mM imidazole (pH 7.4) at -80°C until use.

**Fig. 1:**
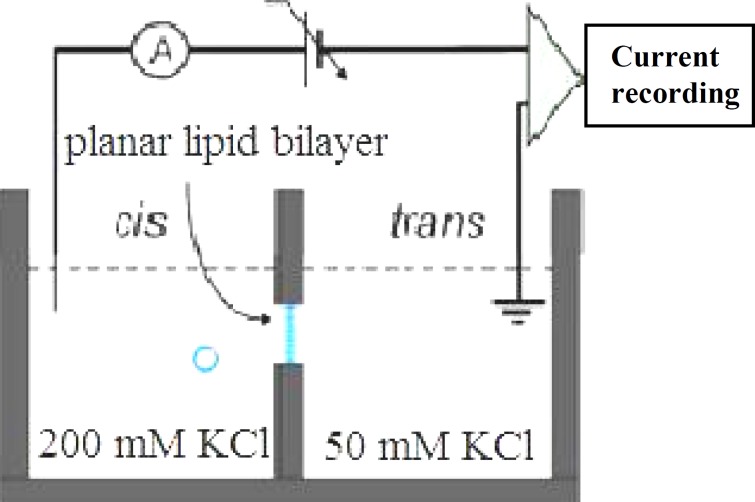
Configuration of the *cis* and *trans* faces. The *cis* chamber (cytoplasmic face) was voltage-clamped relative to the *trans* chamber (luminal face), which was grounded

***Planar lipid bilayers and vesicle fusion*** ***.*** Experiments were performed by using black (bilayer) lipid membrane technique [22]. Planar phospholipid bilayers were formed in a 300 µm-diameter hole drilled in a Delrin partition, which separated two chambers, *cis* (cytoplasmic side) and *trans* (luminal side). Chambers contained 4 ml KCl 200 mM *cis*/50 mM *trans*. Under these conditions, there will be a net movement of water across the bilayer from *trans* to *cis* face. Vesicles in the pre-fusion state will swell if water enters the lumen across the bilayer [22, 23]. *Cis* and *trans* solutions contained 10 µM Ca^2+^. The pH on both sides was adjusted to 7.4 with Tris–HEPES. Planar phospholipid bilayers were painted using a suspension of L-*α*-lecithin in Decane at a concentration of 25 mg/ml. The indication of the thickness of the bilayer membrane formed across the hole was obtained by monitoring capacitance. A low frequency (1-10 Hz) and a low amplitude (5-20 mV peak-to-peaks) triangular wave were used. Typical capacitance values ranged from 200 to 300 pF. Fusion of the vesicles was initiated mechanically by gently touching the bilayer from the *cis* face using a small stainless steel wire of 150 µm diameter, on the tip of which a small drop of the vesicle-containing solution was deposited (Fig. 1).

***Electrical recording and data analysis. ***BC-525D amplifier (Warner Instrument, USA) in the voltage clamp mode was used to amplify the current and also to control the voltage across the bilayer through Ag/AgCl electrodes. The *cis* electrode was set to a command voltage relative to the *trans* electrode which was grounded. The recordings were filtered at 1 kHz with a four-pole Bessel low-pass filter, digitized at a sampling rate of 10 kHz and stored on a personal computer for off-line analysis by PClamp9 (Axon Instruments Inc, USA). The results were expressed as means ± standard error of the means (SEM).

## RESULTS

***Serum glucose concentration. ***Hyperglycemia occurred within 2 week after the injection of STZ. The fasting serum glucose levels (125 ± 7.1, 293 ± 23 and 315 ± 16 mg/dl) were exhibited before the injection, 7 and 14 days after the injection of STZ, respectively, that were elevated significantly compared to control rats 113 ± 7, 120 ± 5, 117 ± 3 mg/dl (n = 48 rats). Therefore, hyperglycemia confirmed diabetic model.

**Fig. 2:**
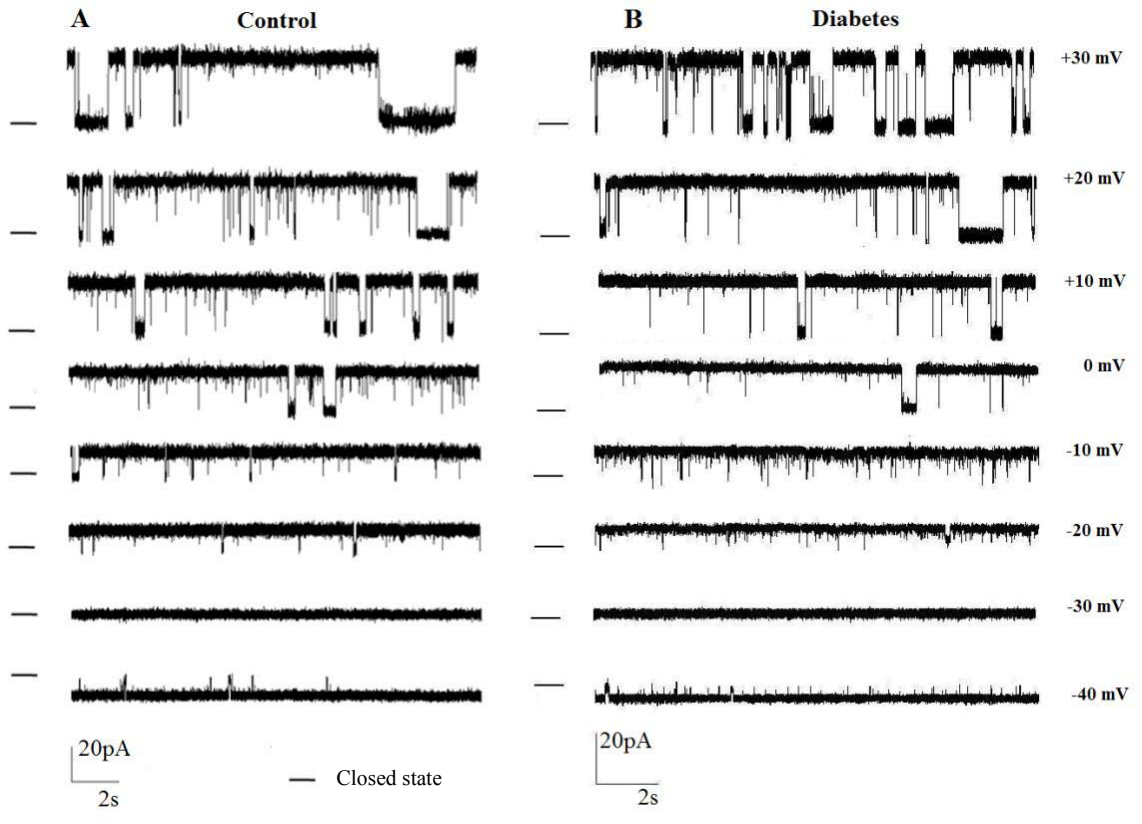
Single-channel recordings as a function of voltages. Single-channel recordings in 200/50 mMKCl (*cis*/*trans*) gradient after reconstitution of liver rough endoplasmic reticulum membrane vesicles in planar lipid bilayer at potentials ranging from +30 to -40 mV in control **(A) **and diabetic **(B)** condition. The – indicates the closed state.

**Fig. 3:**
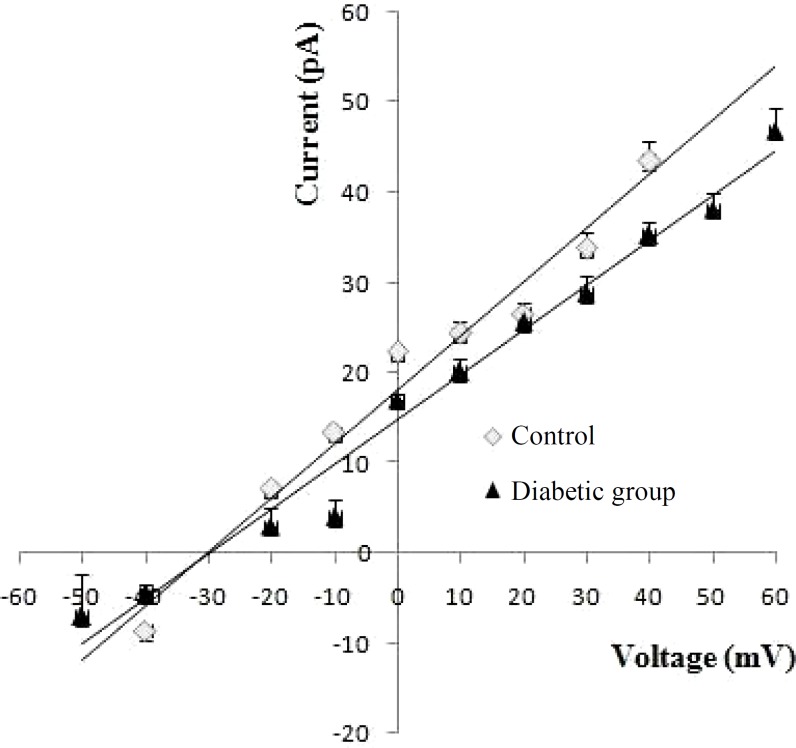
The comparison of single-channel current voltage relationships between diabetes and control. Data points are mean ± s.e.m., obtained from five experiments

***Biophysical properties of ion channel. ***We previously reported the electrophysiological properties of a 598 ± 20 pS potassium channel in endoplasmic reticulum membrane when the *trans* chamber was voltage-clamped relative to the *cis* chamber, which was grounded [10]. In the present study, single-channel recording with voltage-clamped *cis* chamber (next generation of amplifier) was applied to demonstrate whether the gating behavior of this channel is altered under control and diabetes conditions. Figure 2 shows single-channel currents recorded at various holding potential conditions (50/200 mM KCl *trans/cis*) at various holding potentials (-40 mV to +30 mV) following incorporation of rat endoplasmic reticulum membrane vesicles into planar bilayers in control and diabetic rats, respectively. These observations demonstrated a significant increase in the current amplitude for applied positive potentials to -40 mV. In addition, a zero-current potential value close to -34 mV, the equilibrium potential expected for potassium ions under the prevailing ionic conditions was indicated. In addition, the reverse potential close to -34 mV showed unidirectional reconstitution of the channel into lipid bilayer membrane. The channel gating behavior was voltage dependent with decreased channel opening separated by longer silent periods at increasingly positive potential values. As illustrated in Figure 3, the current-voltage (I–V) relation was linear in control and diabetic rats, and the slope conductances were 569 ± 18.5 pS and 520 ± 19pS, respectively with negative reversal potentials close to -30 mV, which attest its cationic selectivity under these conditions. There were no significant differences in channel conductance and current amplitude between control and diabetic rats. 

**Fig. 4:**
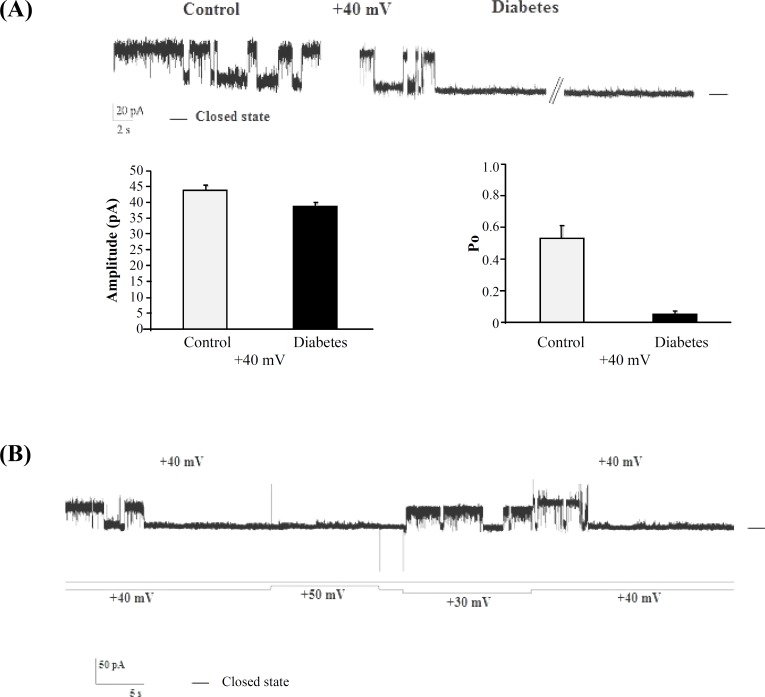
Single-channel recording at +40 mV in control and diabetic conditions. **(A)** Single-channel recording at +40 mV in control (~20 second recording) and diabetic conditions (~3 minutes recording). Significant differences in the open probability value and amplitude are not observed (n = 5). Summarized data show current amplitudes and open probability of reconstituted channels in control and diabetic conditions at +40 mV. Significant differences in the open probability value but not current amplitude are observed (n = 5). **(B)** Current record (above) and applied voltage protocol (below) in diabetic rats. Re-openings of the channel can be observed when the +40 mV was switched to +30 mV in the same bilayer lipid membrane. The – indicates the closed levels

The effect of voltage on the channel activity was investigated by measuring the channel open probability (Po) as a function of voltage. Table 1 shows average steady-state open probability values as a function of the holding potential for full open conducting state obtained from five different experiments in control and diabetic conditions. Open probability of the cationic channel between diabetic and control rats became significantly different at voltages above +30 mV. Indeed, at +40 mV, channel opening was more robust in control rats, and there was a significant reduction in channel Po in diabetic rats. Figure 4A and Table 1 show that Po was 0.53 ± 0.2 in control rats and in diabetic rats it was significantly reduced about 0.05 at +40 mV. Figure 4B shows re-openings of the channel when the +40 mV was switched to +30 mV in the same bilayer lipid membrane. 

**Table 1:** The average steady-state of open probability values as a function of the holding potential for full open conducting state obtained from five different experiments in control and diabetic conditions

**Voltage** **(m** **V)**	**open probability (Po) Control**	**open probability (Po) Diabetes**
+60	0.12 ± 0.07	0^***^
+50	0.20 ± 0.10	0^***^
+40	0.53 ± 0.20	0^***^
+30	0.73 ± 0.10	0.66 ± 0.10
+20	0.83 ± 0.05	0.80 ± 0.06
+10	0.81 ± 0.10	0.81 ± 0.08
0	0.90 ± 0.04	0.88 ± 0.06
-10	0.90 ± 0.08	0.90 ± 0.01
-20	0.93 ± 0.01	0.94 ± 0.04
-40	0.90 ± 0.09	0.94 ± 0.10
-50	0.91 ± 0.08	0.94 ± 0.04

*** *P*<0.001 different from control group

**Fig. 5:**
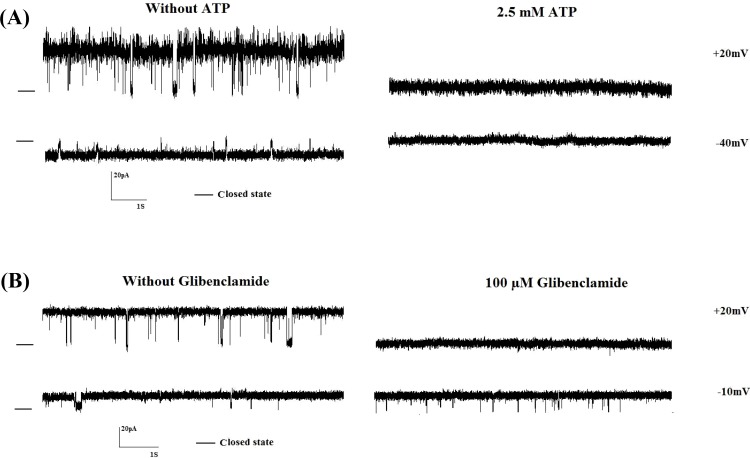
The effect of ATP and glibenclamide on channel activity at different voltages in diabetic rats. Representative recordings of channel currents in the absence or presence of 2.5 mM ATP (A)or 100 µM glibenclamide (B) to *cis* face. Channel activities were completely inhibited after the addition of ATP (n = 5), whereas channel activity was completely blocked at +20 but not -10 mV (n = 5). Closed levels are indicated by –.

***Pharmacological properties of the ion channel. ***Since we focused on ATP- and voltage-sensitive cationic channel in endoplasmic reticulum under diabetic conditions, we determined the pharmaco-logical properties of the channel using ATP and glibenclamide on the channel behavior in diabetic rats. Figure 5A shows single-channel recordings at +20 and -40 mV after addition of 2.5 mM ATP to the *cis *face in diabetic condition. Addition of 2.5 mM ATP totally blocked the channel activities (n = 5). Also, the effect of glibenclamide, as well-known sulfonylurea, to block ATP-sensitive K^+^ channel was examined on channel activity [24]. Figure 5B presents single-channel recordings after addition of 100 µM glibenclamide to the *cis* face (n = 5) at +20 and -10 mV. Glibenclamide (100 µM) blocked channel activities at positive but not negative potentials.

## DISCUSSION

Diabetes as one of the most prevalent and serious metabolic diseases interferes with cell constituents, namely mitochondria and endoplasmic reticulum. Evidence has been shown that endoplasmic reticulum dysfunction contributes to diabetic complications [7]; but the cellular mechanisms remain to be clarified. In this study, we compare some single-channel properties of endoplasmic reticulum cationic channel between diabetic and control rats. Our results showed that channel open probability but not channel conductance is significantly changed in early diabetes.

Using the cell imaging and immunohistochemistry techniques, potassium channels such as K_ATP_ and Ca^2+^-activated potassium channels and their subunits were revealed to be localized on endoplasmic reticulum membrane of cardiomyocytes, neurons, liver, and a muscle cell line (C2C12) [25-27]. Our pervious finding by single-channel technique provided electrophysiological evidence for the potential presence and gating properties of a cationic channel in endoplasmic reticulum of rat hepatocytes [10]. Furthermore, we found that this channel is sensitive to ATP and ADP [21]. It was also reported that the activity of several BK channels was altered by intracellular ATP [28]. In addition, our previous experiment showed that ATP inhibited brain mitochondrial BK channel activity [29]. Therefore, we did not know if the endoplasmic reticulum cationic channel belonged to K_ATP_ channel or BK channel families. Very recently, we pharmacologically characterized K_ATP _channels and described the subunit composition of K_ATP_ channels in endoplasmic reticulum of rat hepatocytes (data has been submitted). Also, there is some evidence that show the K_ATP_ channel is inhibited by sulfonylurea [30, 31]. Figure 5 demonstrates that the addition of 2.5 mM ATP or 100 µM glibenclamide to the cytoplasmic side (*cis* chamber) completely blocks channel activity. According to this result and our previous experiments, this endoplasmic reticulum cationic channel may be a K_ATP_ channel [21]. Furthermore, our result showed that glibenclamide inhibited the channel activity at positive potentials, but unitary current amplitude and gating behavior of the channel were not affected at negative potentials. It has been suggested that glibenclamide does not directly block the channel may bind to either the voltage gate and/or to the inner mouth of the channel [32]. Channel inhibition by glibenclamide is rapidly reversed by elevating the concentration of readily permeate ions in the *trans* solution. Occupation of a binding site with which the gate interacts by K^+^ derived from the *trans* solution would simultaneously reverse the effect of glibenclamide and the voltage-induced block [32]. As mentioned in Methods section, *cis* and *trans* solutions contained 10 µM Ca^2+^, but changes of channel activity were not observed after the addition of 1 mM ethylene glycol tetraacetic acid (data not shown). 

In control and diabetic conditions, the endoplasmic reticulum cationic channel’s I-V curves were linear with a conductance of 569 pS and 520 pS within the range of -40 mV to +30 mV, and no significant differences were observed. The study of Shimoni *et al.* [33] and McGahon *et al.* [34] demonstrated that no significant change was observed in unitary channel conductance of K_ATP_ channels in control and diabetic heart cells. In contrast, there is an evidence that shows potassium current amplitude is decreased in diabetic condition [35]. In this study, although we considered the channel gating behavior in two-week diabetic rats (early diabetes); however, further studies are needed about two-months diabetic rats.

Another observation provided by this study is that endoplasmic reticulum cationic channel is inactivated at lower positive potentials in diabetic rats. We observed that the cationic channel Po is decreased at positive holding potentials to reach a maximum of 0.53 ± 0.2 at +40 mV in control conditions. Interestingly, channel inactivation (Po reaches to 0) was observed in diabetic rats at voltages above +30 mV. These findings are in line with other observations regarding the potassium channel dysfunction in metabolic disease. For instance, Lu *et al.* [36] demonstrated the reduction of mean open times and prolonged mean closed-time durations of BK channel in coronary arterial smooth muscle cells in Zucker diabetic fatty rats. Additionally, reduction of kinetic properties and molecular composition of Kv, K_ATP_, and BK channels as well as decrease of potassium current in plasma membrane of different tissues were consistently observed in diabetic condition [14, 15, 35]. 

In this study, we provide evidence that endoplasmic reticulum cationic channel shows altered gating behaviors. These channel gating properties suggest that cationic channel dwells in longer open states in control rats, but in diabetic rats, they have more frequent inactivation. Both sarcoplasmic reticulum/endoplasmic reticulum Cl^-^ and K^+^ channels act as counter transport systems during rapid Ca^2+^ release and uptake to keep the electrochemical force on Ca^2+ ^ions by maintaining the sarcoplasmic reticulum membrane potential away from E_Ca _[27]. Given that Ca^+2^ regulations by the endoplasmic reticulum is prominent in cellular apoptosis [37, 38], these data suggest that endoplasmic reticulum cationic channel may be involved in the regulation of endoplasmic reticulum-mediated cellular mortality. Moreover, a defect in this regulatory process is the cause of the progressive cell loss and degeneration associated with the disease. Therefore, more research is required to be established via proteomic or via other studies to identity and structure-function relationship of these channel proteins in endoplasmic reticulum. 
